# Olfactory Dysfunction in Familial and Sporadic Parkinson's Disease

**DOI:** 10.3389/fneur.2020.00447

**Published:** 2020-05-29

**Authors:** Bruce A. Chase, Katerina Markopoulou

**Affiliations:** ^1^Department of Biology, University of Nebraska at Omaha, Omaha, NE, United States; ^2^Department of Neurology, NorthShore University HealthSystem, Evanston, IL, United States; ^3^Department of Neurology, University of Chicago, Chicago, IL, United States

**Keywords:** olfactory dysfunction, genetics, idiopathic Parkinson's disease, longitudinal studies, biomarker, cognition, monogenic Parkinson's disease, neurodegeneration

## Abstract

This minireview discusses our current understanding of the olfactory dysfunction that is frequently observed in sporadic and familial forms of Parkinson's disease and parkinsonian syndromes. We review the salient characteristics of olfactory dysfunction in these conditions, discussing its prevalence and characteristics, how neuronal processes and circuits are altered in Parkinson's disease, and what is assessed by clinically used measures of olfactory function. We highlight how studies of monogenic Parkinson's disease and investigations in ethnically diverse populations have contributed to understanding the mechanisms underlying olfactory dysfunction. Furthermore, we discuss how imaging and system-level approaches have been used to understand the pathogenesis of olfactory dysfunction. We discuss the challenging, remaining gaps in understanding the basis of olfactory dysfunction in neurodegeneration. We propose that insights could be obtained by following longitudinal cohorts with familial forms of Parkinson's disease using a combination of approaches: a multifaceted longitudinal assessment of olfactory function during disease progression is essential to identify not only how dysfunction arises, but also to address its relationship to motor and non-motor Parkinson's disease symptoms. An assessment of cohorts having monogenic forms of Parkinson's disease, available within the Genetic Epidemiology of Parkinson's Disease (GEoPD), as well as other international consortia, will have heuristic value in addressing the complexity of olfactory dysfunction in the context of the neurodegenerative process. This will inform our understanding of Parkinson's disease as a multisystem disorder and facilitate the more effective use of olfactory dysfunction assessment in identifying prodromal Parkinson's disease and understanding disease progression.

## Introduction

Since Ansari and Johnson (1) first reported that olfactory dysfunction (OD) occurs in Parkinson's disease (PD), OD has been evaluated using tests of odor identification, odor discrimination, odor-threshold detection and electrophysiology (2–4). OD is not PD-specific and is prevalent in aging and other diseases, particularly in neurodegenerative disorders such as Alzheimer's disease, Huntington's disease, and rapid-eye-movement sleep-behavior disorder (5–12). OD can severely impact the quality of life, affecting interpersonal and eating habits, patient safety, and nutritional intake (13–15). Because OD is prominent in PD (16, 17) and its onset may signal prodromal PD, it is important to understand how and when OD arises, the mechanisms underlying its association with PD progression, and identify interventions for OD.

## OD Prevalence in PD

Cross-sectional studies revealed that OD occurs in sporadic PD prior to the initiation of dopaminergic therapy [reviews: (3, 4, 18–20)]. The reported prevalence of OD in sporadic PD varies substantially: 45–50% (1, 21, 22), 70–80% (2, 23), and 90–97% (24, 25). This may reflect challenges in PD diagnosis, OD measurement, sample size, normative group selection, and age. Prevalence of OD generally decreases when adjusted for age-related norms, as the prevalence of OD is over 50% past age 65 and 62–80% past age 80 (26, 27).

**Figure 1:**
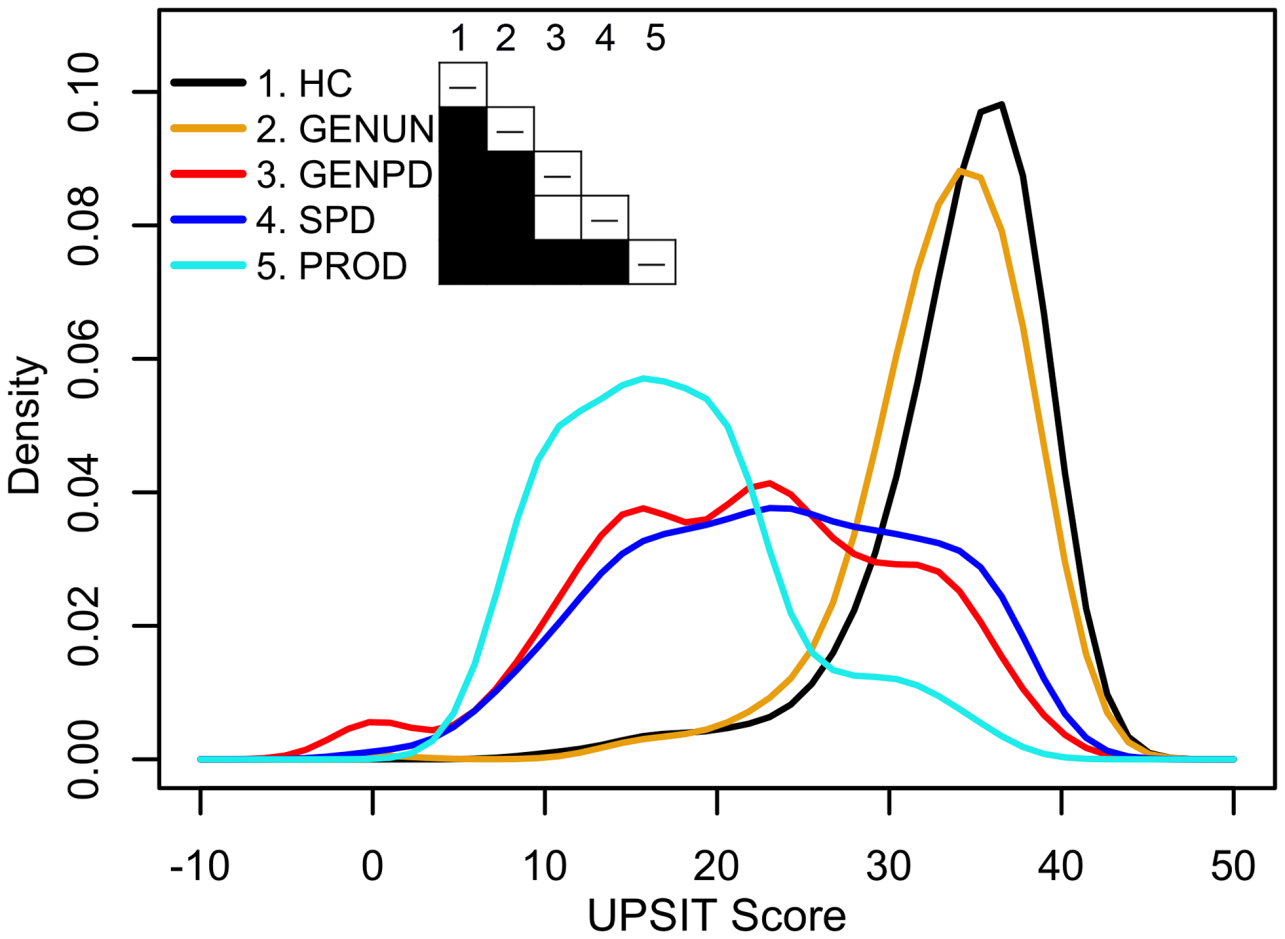
Univariate density estimates of scores on the University of Pennsylvania Smell Identification Test (UPSIT) in five PPMI cohorts (69). Cohorts: 198 healthy controls (HC, black) age-matched with 491 sporadic Parkinson's disease patients (SPD, blue, ≥2 of resting tremor, bradykinesia, or rigidity, with resting tremor or bradykinesia required, or either asymmetric resting tremor or asymmetric bradykinesia; PD diagnosis ≤2 years; Hoehn and Yahr stage I–II; scan-confirmed dopaminergic deficit; ≥30 years at diagnosis; no dopaminergic medications ≥6 months after baseline assessment), 310 asymptomatic genetic Parkinson's disease patients who have a mutation, or are a first-degree relative of an individual having a mutation, in LRRK2, SNCA, or GBA (GENUN, gold), 220 symptomatic genetic Parkinson's disease patients who have a mutation in LRRK2, SNCA, or GBA (GENPD, red), and 61 individuals selected for REM-behavior sleep disorder and/or hyposmia (PROD, cyan). Shading in the table cells indicates the *P*-value (white: *P* ≥ 0.05, black: *P* < 0.001) obtained from pairwise non-parametric bootstrap tests of equal densities using 1,000 permutations.

Interestingly, OD in monogenic PD exhibits variable penetrance and expressivity. In manifesting carriers with *GBA* (β-glucosylceramidase), *SNCA* (α-synuclein, point or gene-multiplication), *LRRK2* (leucine-rich repeat kinase 2), *PINK1* (PTEN-induced kinase 1), or *DJ1* (PARK7: Parkinsonism-associated deglycase) mutations, and in *MAPT* (microtubule-associated protein tau)-associated frontotemporal dementia and parkinsonism, OD-penetrance overlaps with that in sporadic PD [(28–64); reviews: (3, 65–68)]. While different studies report varying, sex- or allele-differential OD prevalence in mutation carriers relative to sporadic PD controls [tabulated in Doty (3)], two key OD features seen in sporadic PD persist in many monogenic forms. First, while many carriers are hyposmic when they phenoconvert to show motor symptoms, some carriers have mostly preserved olfaction (28, 40). Second, the distribution of OD in monogenic PD cohorts is similar to sporadic PD (Figure 1). The striking exception is *PRKN* (parkin RBR E3-ubiquitin protein ligase) and *VPS35* (VPS35 retromer-complex component) manifesting carriers, who have normal olfaction or only mild OD (70–75). As discussed below, the preserved olfaction in *PRKN* carriers and possibly some subsets of *LRRK2* carriers appears related to an absence of Lewy bodies (LBs) in the olfactory bulb and/or the olfactory system (76–78).

Mutations in *LRRK2, PINK1, GBA, SNCA*, and *PRKN* have similar effects on OD across ethnically and geographically diverse populations. Hence, if a mutation causes OD, its effect-size on OD-related neurodegenerative processes is large relative to genetic background and environmental exposure. Since these mutations increase substantially PD risk, targeted investigations of non-manifesting mutation carriers of *LRRK2, PINK1, GBA*, and *SNCA* provide a unique opportunity to understand OD in PD.

## OD Characteristics in PD

Though many fundamental questions about OD in PD have been raised for some time (24, 79, 80) and studied in diverse patient cohorts and contexts, consensus answers are not always available, as described below. Sometimes, conflicting findings reflect the tests used or their interpretation. As discussed more fully by Doty (81), while the results on psychophysical tests of OD (tests of odor identification, odor discrimination, or odor-threshold detection) are strongly correlated, they vary in reliability and sensitivity and assess different neurophysiological, neurological, and/or psychological aspects. Most often, OD is evaluated using tests of odor identification. Using those tests, variable OD is seen in all studies of sporadic PD and those monogenic PD forms resembling sporadic PD (*SNCA, GBA, LRRK2*), including at motor-symptom onset. Figure 1 (69) illustrates this using univariate density estimates of odor-identification-test scores obtained from the Parkinson's Progression Marker Initiative (PPMI). While the score distributions of early-stage, dopamine-transporter-scan positive, dopaminergic-treatment naïve sporadic PD (blue), and age-matched healthy controls (black) are distinct, both groups have normosmic, hyposmic, and anosmic membership. This is also observed in manifesting *SNCA, GBA*, and *LRRK2* carriers (red line), which here have a score distribution like sporadic PD. Indeed, anosmia is not always seen in manifesting carriers in nuclear families with monogenic PD (28, 52). Hence, like PD motor symptoms, OD has variable penetrance in sporadic and some monogenic PD. Unlike them, OD is frequently seen in otherwise healthy aging and other neurodegenerative diseases, suggesting that OD can result from a confluence of processes.

Though OD in PD presents non-uniformly, community-based prospective studies demonstrated that it can appear up to 4 years before motor-symptom onset (79, 80); in MAPT carriers it can appear 2 years before symptom onset (52). Consequently, OD has been used in biomarker panels for predicting risk and/or progression of PD [(59, 82–88); reviews: (18, 19, 89–91)]. For this purpose, it is important to elucidate: (1) whether OD in PD is distinguishable from OD in other diseases and aging; (2) how its onset and progression relates to motor-symptom onset and progression; (3) whether OD severity is associated with disease stage, duration, or predicts disease progression; and (4) what clinical tests of OD measure in the context of the disease process. Well-designed studies of OD in monogenic PD can address each issue.

## Distinguishing Features of OD in PD

Central to understanding whether the etiology of OD in PD is shared with that in the elderly or other neurodegenerative diseases is identifying whether OD has PD-specific characteristics. PD does affect supra-threshold estimates of perceived odor intensity, which appears spared in Alzheimer's disease, schizophrenia, and the elderly (92), but does not affect the trigeminal system (93). Combined with imaging, it can help distinguish disorders whose initial presentation overlaps with PD, such as progressive supranuclear palsy, cortico-basal degeneration, or multiple system atrophy [(94–96); reviews: (20, 97)].

Many studies have identified a set of odors or pattern of OD that best evaluates OD in their cohort (98–107). Most often however, the odor sets are dissimilar in different PD populations (108). This likely reflects odor identification being influenced strongly by prior exposure and population variation in odorant-receptor alleles. Multiple analyses have indicated that there is not odor-selective hyposmia in PD. Highly compelling is an odor-item analysis indicating that the discriminatory power of odor subsets is not shared across independently selected groups (109). Additional support comes from longitudinally evaluating hyposmia in subjects with sporadic PD, subjects without neurodegenerative disease, and in *MAPT-*mutation carriers. They reveal odor-identification irreproducibility as a general feature of OD: subjects do not misidentify the same odors on replicate odor-identification tests (52). In a longitudinal study of sporadic or monogenic PD subjects recruited from ethnically diverse populations, comparison of results across populations would be facilitated by using a universal olfactory test that is independent of odor-specific insensitivity or prior experience (110).

## How Is OD Related to Disease Onset and Progression?

The etiological mechanisms underlying the variable presentation of both PD motor symptoms and OD remain unclear. The olfactory epithelium in PD appears normal (111), but it is unknown whether PD impacts its neurogenic niche (112), the functional integration of axons from differentiating olfactory-receptor neurons into the olfactory bulb, and how either process impacts OD. α-Synuclein deposits are found in the olfactory bulb and anterior olfactory nucleus at Braak stage I (113–115), and glomerular volume is reduced by half in PD (116). Since the olfactory bulb plays a critical role in the spatiotemporal coding of smell, OD early in disease might reflect the incomplete inhibition of olfactory inputs at the level of the olfactory bulb (117) and the reported increase in dopaminergic neurons (118, 119). Studies of OD in monogenic PD offer a compelling hypothesis for the variable expressivity of OD: early OD reflects LB development in the olfactory bulb. LBs are prominent neuropathological features in monogenic PD forms with OD (*SNCA, GBA, PINK1*, and *DJ1*), but not in *PRKN-*related PD where olfaction is preserved (28–64, 70–77). Progressive OD is also seen in mice expressing forms of human α-synuclein exhibiting olfactory-bulb Lewy pathology (78, 120, 121). *ATP13A2* (ATPase cation transporting 13A2) carriers exhibit OD (65) but not LB (122), but show atypical PD. Since LB and olfactory dysfunction are not always seen in *LRRK2* carriers, and some *LRRK2* alleles have fewer LB (40–49, 76, 77, 123), additional support for this hypothesis would come if the relatively preserved olfaction in a subgroup of *LRRK2* carriers were also associated with fewer olfactory-bulb LB. If this hypothesis is correct, screening hyposmic individuals using PET ligands under development to image LB in the olfactory bulb (124)^1^ would help identify those having increased risk of developing PD-motor symptoms.

**Figure 2:**
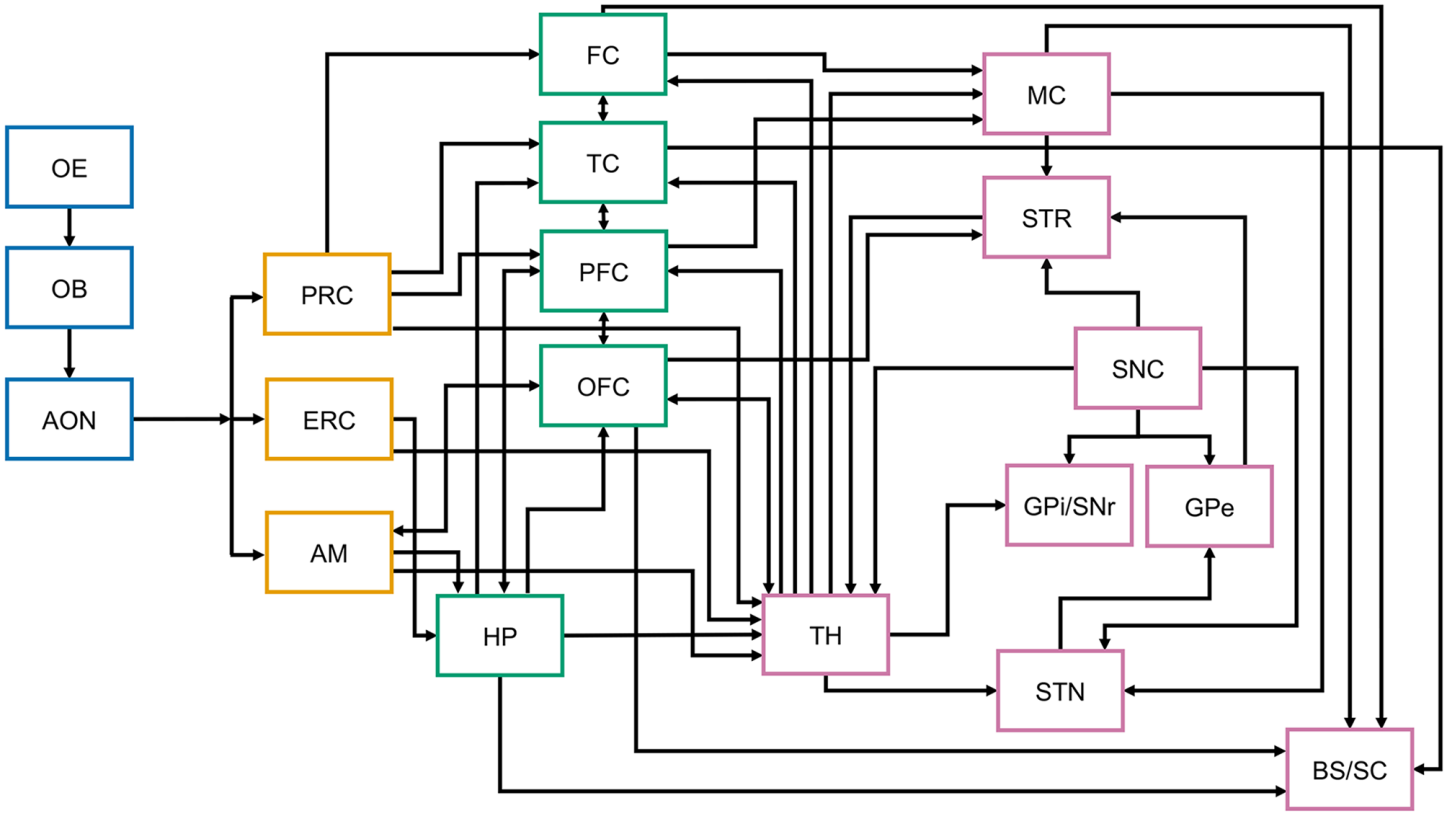
Simplified schematic representation of central nervous system structures and connections involved in olfaction, memory, and motor control. The figure aims to illustrate the complexity of the connections of the olfactory system, associative cortices, thalamus, and the basal ganglia that may be differentially affected at different stages of Parkinson's disease. While the arrows represent anatomical and functional connectivity, not all known interconnections are included in this schematic representation. Differential neuronal loss and associated decrease in key neurotransmitter (acetylcholine, dopamine, etc.) levels at any of these structures has the potential to differentially affect their function and connectivity, thus directly and indirectly contributing to olfactory dysfunction. While in PD LB preferentially involve the brainstem at disease onset, their distribution in the olfactory and cortical areas depends on disease stage (113, 114). OE, olfactory epithelium; OB, olfactory bulb; AON, anterior olfactory nucleus; PRC, perirhinal cortex; ERC, entorhinal cortex; AM, amygdala; FC, frontal cortex; TC, temporal cortex; PFC, prefrontal cortex; OFC, orbitofrontal cortex; HP, hippocampus; TH, thalamus; MC, motor cortex; STR, striatum; SNC, substantia nigra pars compacta; GPi/SNr, globus pallidus interna/substantia nigra pars reticulata; GPe, globus pallidus externa; STN, subthalamic nucleus; BS, brainstem.

Early olfactory deficits are consistent with the olfactory vector hypothesis for PD pathogenesis and the caudo-rostral spread of LB pathology (113–115). It is interesting however, that some individuals with normal olfaction lack olfactory bulbs (125). This suggests that the establishment and maintenance of olfactory circuits has considerable functional plasticity. The projections of the olfactory tract form circuits spanning multiple cortical areas, including the entorhinal and orbitofrontal cortices and utilize multiple neurotransmitter systems (Figure 2). Therefore, olfactory-bulb pathology may not be the sole determinant of OD. As discussed below, early OD associated with olfactory bulb LB can be followed by later cholinergic denervation (126, 127). It will be important to address the extent to which OD in PD is associated with a loss of functional plasticity, whether it reflects the differential progression of the neurodegenerative process in one or multiple anatomical regions, the contributions of degenerative or compensatory changes in dopaminergic, or other neurotransmitter systems, including substance P and acetylcholine (128, 129), and how these associations relate to later motor-symptom onset and progression.

OD has significant, moderate to strong associations with nigrostriatal degeneration (105, 130, 131). In one study, 98.7% of PD subjects with imaging evidence of nigrostriatal dopaminergic denervation had OD (130). There, however, most still retained some olfactory function: 24.6% were anosmic and 73.2% were hyposmic [*N* = 183, motor-disease duration = 6.4 ± 4.3 year, Hoehn and Yahr (H&Y) stage 1–5]. Consistent with these findings, screens for hyposmia increase the likelihood of identifying subjects with abnormal dopamine transporter binding (91). This may be a causal association or reflect coincident processes. Deficits in cholinergic transmission are a common element in OD in different diseases (132), and neurodegeneration affecting cholinergic circuits is found even early in PD [Figure 2, (133–136)]. Indeed, cholinergic denervation of the limbic archicortex in PD subjects at H&Y 2.5 ± 0.5 is a more robust determinant of poor odor-identification test scores than nigrostriatal dopaminergic denervation (126). When groups of PD patients having mild motor deficits and varying degrees of OD were compared, there was a more significant reduction of a putative cholinergic marker (i.e., short latency afferent inhibition of the motor cortex) when olfactory event-related potentials (a direct measure of the processing of olfactory information) were absent, than when only their latency and/or amplitude was altered (137). Curiously, a history of smoking (cholinergic stimulation) is also associated with better olfaction in PD (138).

The use of shared neural substrates in the premotor frontal and orbitofrontal cortex by olfaction and cognition (Figure 2), and the contribution of cholinergic deficits to OD provides a potential mechanism for why greater OD appears to identify the subset of sporadic and monogenic PD patients at greater risk of future cognitive impairment [(59, 107, 139–149); reviews: (27, 150)]. Thus, genome-wide screens in PD subjects for variants that influence risk of severe OD or protect from developing OD may identify genetic factors that increase risk of, or offer protection from, cognitive impairment in PD. PD-associated changes to central brain networks, brain-region specific structural integrity, and functional connectivity also are associated with OD (151–161). The importance of functional connectivity is highlighted by theta-specific phase coupling between the piriform cortex and hippocampus in the rapid differentiation of odor stimuli (162), and the ability of anosmic subjects having diminished functional connectivity to activate an olfaction-related functional network (163). A possible partial restoration of functional connectivity may explain why deep-brain stimulation of the subthalamic nucleus (DBS-STN) leads to modest odor-identification test score improvement (164–166). To obtain mechanistic insights into the variable presentation of OD, its relationship to motor symptom presentation and later cognitive dysfunction, it would be fruitful to longitudinally study carriers of monogenic PD mutations utilizing functional imaging to evaluate how functional connectivity is altered during prodromal PD and disease progression.

Since olfactory system LB increase with advancing neuropathological PD stage (113–115) and many non-motor PD symptoms such as cognitive and autonomic dysfunction often increase in severity with disease progression, it is unclear why the severity of hyposmia is not consistently associated with motor signs, disease stage, or duration. This is especially striking because the density of synuclein-pathology in the olfactory bulb is positively correlated with motor scores (165). Some cross-sectional studies reveal that diminished scores on olfactory-function tests are associated with increased disease duration (167, 168), while others do not (22, 23, 25, 117, 148, 169, 170). Some studies have reported associations with more severe disease (22, 148, 168, 169, 171, 172) but others have not (24, 25, 117, 170, 173–175), even though hyposmia severity is associated with lower dopamine transporter activity (168). While OD does not always develop in parallel with other non-motor symptoms in either sporadic or monogenic PD (65), resolving whether it does develop in parallel with motor symptoms has implications for management. In one study of PD subjects with similar striatal dopamine transporter activities, normosmic individuals had lower levodopa-equivalent dose requirements than did hyposmic individuals at 2.5 years of follow-up (22), suggesting that a relative lack of OD may be associated with a clinically more benign disease course.

The conflicting results about whether OD relates to disease progression might be explained if OD does not appear gradually, but rather in a stepwise irreversible manner. Variability in the occurrence of LB within the olfactory bulb could be related to the degree of inhibition of olfactory inputs (117) and/or increase in dopaminergic neurons (118). This could contribute to variable expressivity in initial OD that remains relatively stable over time, possibly due to functional plasticity. Stepwise onset could arise from the convergence of multiple failing processes. While a primary early contributor is almost certainly the loss of functionality within the olfactory bulb, later contributions could derive from other olfactory-system regions. These could include the asynchronous stepwise failure of compensatory mechanisms and/or the onset of dysfunction in circuits involved in associative processing and interpretation of smell. Joining the gradual loss of functionality in the olfactory bulb to either of these processes would lead to a stepwise onset of OD in PD. In this scenario, different levels of OD would be observed upon breaching different functional lintels. A continuous scaled-test score distribution would be observed in a population, but longitudinally followed individuals would show stepwise score decline. Since many newly diagnosed cases are normosmic or hyposmic, whether or not an association is observed between OD and motor function in a cross-sectional study would depend strongly on the cohort's initial constitution.

Whether OD shows stepwise progression could be addressed by obtaining longitudinal data on OD in large PD cohorts. To date, most studies (e.g., PPMI) assess OD only at baseline. Hyposmia can be stable over periods of 2–6 years in sporadic PD (24, 117), *MAPT* mutation carriers (52), and *GBA* mutation carriers (50). Therefore, to assess the progression of OD accurately, follow-up longer than 5 years will be necessary. A more efficient approach is to assess the progression of OD in non-manifesting carriers from monogenic PD cohorts where disease risk is substantially increased, and the genetic cause is known. A longitudinal study using imaging methodologies able to evaluate when LB appear, the integrity of multiple neurotransmitter systems, and functional connectivity would help address the relative contribution of each to the onset and progression of OD and motor symptoms.

## What Do Olfactory-Function Tests Assess About the Disease Process?

The stability of measurements of OD in PD suggests that it may be challenging to use them to directly assess the prodromal and symptomatic disease process outside of monogenic PD cohorts. Intriguingly, PD subjects often subjectively assess their olfactory ability as better than evaluated by validated clinical measures (13, 24, 176–178). One study (176) found 91% hyposmic subjects using the UPSIT, an objective odor-identification test, vs. 55% using a subject's subjective assessment. Lower scores on clinical tests have implications for a patient's quality of life. Patients unaware of their olfactory deficit may be at greater risk of harm because they may be unable to detect smoke or spoiled foods (178). However, this concern may be tempered if the perception of the patient is not fully captured by the objective assessment.

An explanation for the discrepancy between the objective and subjective assessments comes from finding that a loss of awareness of hyposmia is associated with mild cognitive impairment in PD (177). PD patients who overrate their sense of smell or are aware of their hyposmia have worse executive function than those who are objectively and subjectively normosmic (13). Memory is strongly related to olfaction, and deficits in olfaction and verbal learning/memory in PD are associated (107, 126, 179–182). Deficits in cognitive processes also indirectly contribute to lower scores on forced-choice odor-identification tests (69). Consequently, discrepancies in the metacognitive knowledge of hyposmic individuals—self-awareness of their olfactory ability—and objectively measured OD may reflect testing-related cognitive challenges in memory or decision making. This lack of metacognitive knowledge may be a sensitive biomarker of early cognitive decline (13). A lack of metacognitive knowledge may also identify individuals whose olfactory system can have functionality restored. If a subject's perceptual reality is better than their objectively assessed ability, some of the neural substrates used for processing olfactory information should be preserved. Assessing metacognitive knowledge within longitudinal studies of monogenic PD could help identify the neural substrates preserved when metacognitive knowledge does not match objective measurements, and which are lost when individuals self-perceive anosmia. This has pragmatic considerations for managing cognitive decline.

Identifying individuals whose olfactory system could have functionality restored also identifies candidates for potential OD therapy. While motor symptom treatment is a primary concern in PD, improving non-motor symptoms like OD will improve patient quality of life (13, 183). Simple strategies to improve OD are lacking presently. While DBS-STN modestly improves OD (163–166), DBS-STN is currently used to treat motor complications of levodopa therapy in patients with an at-least 4 year disease duration. It will be informative to assess if other treatments currently under development, such as α-synuclein antibody therapy, gene-editing therapy or other molecular treatments specific to monogenic forms of PD, also have a beneficial effect on OD.

## Conclusion

Elucidation of the mechanisms underlying OD in PD and their relationship to the onset and progression of motor and cognitive symptoms will contribute to comprehensive measures of OD being used to better understand, identify and manage PD. Well-characterized monogenic cohorts identified within the GEoPD and other international consortia (184) can serve as the ideal substrate for multifaceted longitudinal studies needed for this purpose.

## Author Contributions

KM and BC contributed to the conception of the review. BC wrote the first draft of the manuscript. BC and KM contributed to manuscript revision, read, and approved the submitted version.

## Conflict of Interest

The authors declare that the research was conducted in the absence of any commercial or financial relationships that could be construed as a potential conflict of interest. The handling editor declared a past collaboration with the authors.

## Footnotes

^1^https://www.michaeljfox.org/grant/18f-labeled-alpha-synuclein-ligands-pet-imaging-lewy-bodies
